# circSLC30A7 Inhibits Hepatocellular Carcinoma Cell Proliferation via the miR-767-5p/FBXW7/NOTCH1 Axis

**DOI:** 10.1155/2021/8800657

**Published:** 2021-10-12

**Authors:** Xiaotian Yu, Yun Zhang, Chao Jiang, Feng Zhan, Zhenwei Shen, Zhenghai Shen, Feng Zhang

**Affiliations:** ^1^Liver Transplantation Center, The First Affiliated Hospital of Nanjing Medical University, Nanjing, China; ^2^Department of General Surgery, The Affiliated Yixing Hospital of Jiangsu University, Wuxi, China

## Abstract

Circular RNAs, noncoding RNAs, have attracted much attention in various human tumor research fields. They regulate the development of various human cancers via microRNA sponges. This study aimed to assess the molecular mechanism of circSLC30A7 in hepatocellular carcinoma (HCC). In our study, we identified that circSLC30A7 was significantly downregulated in HCC cell lines and tissues. Furthermore, gain and loss function experiments were conducted to elucidate the biological functions of circSLC30A7 in HCC cell lines. Mechanistically, circSLC30A7 sponged miR-767-5p, inhibiting the expression of its downstream protein, FBXW7. In summary, this study revealed that circSLC30A7 is an essential tumor suppressor that inhibits HCC tumorigenesis through the miR-767-5p/FBXW7/NOTCH1 axis. Taken together, circSLC30A7 reduces HCC malignancy and can be a biomarker for HCC management.

## 1. Introduction

Hepatocellular carcinoma (HCC) is a common human malignancy with high prevalence and mortality rates worldwide [1]. Early-stage HCC patients can be treated using surgical treatments, such as liver resection and transplantation. However, the treatment efficacy and 5-year survival rate for HCC patients are poor due to the lack of highly specific and sensitive early diagnostic biomarkers [2, 3]. Therefore, it is necessary to elucidate the molecular mechanisms of HCC and identify the molecular biomarkers for HCC prevention and treatment.

Circular RNA (circRNA), a noncoding RNA, is produced via backsplicing of precursor mRNAs. circRNAs were first discovered in the eukaryotes in 1979 [4, 5]. However, the function of circRNAs is unknown. Several studies have associated circRNAs with many core transcription and termination spliceosomal factors [6, 7]. Numerous other studies have also revealed that circRNAs are associated with the development and progression of various tumors, such as hepatocellular carcinoma [8], bladder tumors [9], breast tumors [10], and thyroid cancers [11]. Recently, circRNAs can act as microRNA or protein sponges [12–15].

In this study, a novel HCC-related tumor suppressor, circRNA, was identified via data mining in three GEO databases (GSE78520, GSE94508, and GSE97332). Hsa_circRNA_100291 was identified at chr1:101372407-101387397 with a spliced sequence length of 660 nt and named hsa_circ_0000098 at Circbase database, which derived from SLC30A7. Besides, the genomic structure analysis demonstrated that hsa_circRNA_100291 was mapped at the 3, 4, 5, 6, 7, and 8 exons of the SLC30A7 gene, thus naming it as circSLC30A7. This study showed that circSLC30A7 expression level was decreased in HCC cell lines and tissues. Furthermore, circSLC30A7 inhibited the proliferation of HCC cells by binding to miR-767-5p as a miRNA sponge to regulate FBXW7 expression.

## 2. Materials and Methods

### 2.1. Patient Enrollment and HCC Specimen

A total of 50 pairs of HCC biopsies and adjacent nontumor tissues were excised from confirmed HCC patients and received surgery at the Department of Surgery of The First Affiliated Hospital of Nanjing Medical University between March 2018 and October 2019. All fresh tissues were stored in liquid nitrogen at −80°C. No patient received radiotherapy or chemotherapy treatment before surgery. The clinicopathological features of PTC patients are shown in Table 1. All participants signed written informed consent before tissue sample analysis. The study followed the provisions of the Declaration of Helsinki and was approved by the Ethics Committee of The First Affiliated Hospital of Nanjing Medical University.

### 2.2. Cell Culture and Transfection

The human HCC cell lines (HepG2, SMMC-7721, Huh-7, and Bel-7404) and control cell HL-7702 were sourced from the Cell Bank of the Chinese Academy of Sciences (Shanghai, China). All cells were cultured using Dulbecco's modified eagle medium (DMEM) (Gibco, Carlsbad, California, USA) with 10% fetal bovine serum (FBS) (Gibico, Carlsbad, California, USA) at 37°C in a humidified atmosphere with 5% CO2. miR-767-5p mimics or miR-767-5p inhibitor (anti-miR-767-5p) and their respective negative controls (miR-NC or anti-miR-NC) were obtained from GenePharma (Shanghai, China). The short hairpin (sh)-negative control (sh-control), sh-circSLC30A7, sh-FBXW7, overexpression (oe)-NC (empty vector), oe-circSLC30A7, and oe-FBXW7 were sourced from Genomeditech (Shanghai, China). Lipofectamine 3000 (Invitrogen, Carlsbad, CA, USA) was used for cell transfection following the manufacturer's guidelines. Further analyses were conducted after 24 h of cell transfection. The circRNA-overexpressed plasmid was constructed by GV486 vector and KpnI/BamHI digestion from Genechem, Shanghai. Firstly, the vector needs to amplify the target circRNA sequence. Then, two specific sequences at the 5′ end and 3′ end and KpnI/BamHI were added.

### 2.3. RNA Extraction and Quantitative Real-Time PCR (qRT-PCR) Assay

The TRIzol reagent (Invitrogen, Carlsbad, CA, USA) was used to extract total RNA from cell and tissue samples. The Cytoplasmic and Nuclear RNA Purification Kit (Invitrogen, Carlsbad, CA, USA) was used to extract the nuclear and cytoplasmic fractions. The SYBR Premix Ex Taq kit (TaKaRa, Japan) was used to detect the mRNA level of target genes via the StepOnePlus™ Real-Time PCR system (Applied Biosystems, USA). The relative expression levels were calculated using the 2^−ΔΔCt^ method. All reactions were conducted in triplicate. The primers are as follows: F: GGTGTAATTGCTTCTGCCATC; R: ATAACAGAAGCTGCCAGTCCA.

### 2.4. RNase R Digestion and Actinomycin D Treatment

HepG2 and Huh-7 cells were extracted from total RNA. Total RNA (3 *μ*g) was incubated with or without 3 U/mg RNase R (Epicentre Biotechnologies, USA) at 37°C for 15 min for qRT-PCR analysis. The culture medium was added to actinomycin D (100 ng/ml) or DMSO (Amresco, USA) to block new RNA synthesis in HepG2 and Huh-7 cells for stability analysis of circSLC30A7 and its linear isoform. The qRT-PCR was used to examine the relative RNA levels in HepG2 and Huh-7 cells at the indicated time points.

### 2.5. Fluorescent In Situ Hybridization (FISH)

Cy3-labeled probes were specific to circSLC30A7 and were obtained from RiboBio (Shanghai, China). A FISH Kit (RiboBio, Shanghai, China) was used to detect the probe signals following the manufacturer's instructions. Nikon AISi Laser Scanning Confocal Microscope (Nikon, Japan) was used for visualization. The probe is as follows: 5′ ATCAAGCCTAAGCTTACAAC 3′.

### 2.6. Cell Proliferation Assays

CCK-8 assay kits, ethynyldeoxyuridine (Edu) assays, and colony formation assays were used for cell proliferation analysis. For the CCK-8 assay, treated cells were cultured in 96-well plates, and CCK-8 reagent (Takara, Nanjing, China) was added at 1, 2, 3, 4, and 5 days. Absorbance quantification was then conducted at 490 nm. Similarly, the cells were incubated in a 96-well plate after transfection for Edu assays. A 20 *μ*M Edu labeling media (KeyGENBioTECH, Nanjing, China) was added to a 96-well plate and then incubated for 2 hours. The cells were treated with 0.5% Triton X-100 and 4% paraformaldehyde, and then an anti-Edu working buffer was used for staining. Fluorescence microscopy was used to visualize the cells, detecting the Edu-positive cells. For colony formation assay, the treated cells were placed into 6-well plates and incubated for 14 days. The cells were then fixed in methanol for 30 min and stained using crystal violet solution.

### 2.7. Cell Apoptosis Determination

Cells were transfected under different conditions for cell apoptosis assay. The cells were washed twice using PBS 48 hours after transfection and then treated with trypsin (without EDTA). The cells were reconstituted in a binding solution, stained with 5 *μ*l of annexin V-FITC (Beyotime, Nanjing, China) reagent, and then incubated in a dark place at room temperature (RT) for 15 minutes. 1 *μ*l of propidium iodide (PI, 50 *μ*g/ml) (Beyotime, China) was added, gently mixed, and then incubated in darkness at RT for 5 min for detection. Flow cytometry (FACScan, BD Biosciences) with the Cell-Quest software was used to analyze the apoptotic cells.

### 2.8. Dual-Luciferase Reporter Assay

The fragments containing the wild type (wt) and the mutant type (mut) of circSLC30A7 and FBXW7 were cloned into the luciferase reporter target expression vector (Promega, China). For circSLC30A7 and miR-767-5p luciferase assays, HepG2/Huh-7 cells were cotransfected with miR-767-5p mimics and circSLC30A7 luciferase reporter. The HepG2/Huh-7 cells were transfected with miR-767-5p mimics and FBXW7-3ʹUTR-luciferase reporter (wt and mut) for miR-767-5p and FBXW7 luciferase assay. Luciferase Assay System (Promega, China) was used for cell detection after 48 h.

### 2.9. Western Blot Assay

Protein extracts were separated through a 10% SDS-PAGE gel and then transferred to a 0.4 mm PVDF membrane. The membranes were treated with 5% nonfat milk at RT for 2 h and then incubated with primary antibodies at 4°C overnight. The membrane was then treated with a secondary antibody. The BeyoECL Plus Kit (Beyotime, Nanjing, China) was then used to visualize the protein band. GAPDH was used as an internal control. The primary antibody used included: rabbit anti-GAPDH, anti-FBXW7, anti-Notch1, anti-CyclinD1, anti-PCNA, anti-Bcl-2, and anti-Bax at 1 : 1000 (Cell Signaling Technology, USA).

### 2.10. Tumor Growth in Nude Mice

HCC cell lines, Huh7 and HepG2, were transfected with vector or circSLC30A7-ov and sh-NC or sh-circSLC30A7, respectively; then subcutaneously injected into the flanks of female athymic specific-pathogen-free (SPF) BALB/C nude mice (aged 4-5 weeks). Mice were sacrificed after 28 days, and then tumor weights were evaluated. The Animal Care and Use Committee of The First Affiliated Hospital of Nanjing Medical University approved the study protocols.

### 2.11. Data Analysis

The SPSS 22.0 software and GraphPad Prism 7 were used for data analyses. Student's *t*-test and chi-squared test were used to assess differences among groups. Pearson correlation analysis was used to determine the relationships among circSLC30A, miR-767-5p, and FBXW7 expressions. *p* < 0.05 represented statistically significant differences.

## 3. Results

### 3.1. circSLC30A7 Is Downregulated in HCC Tissues and Cells

**Figure 1:**
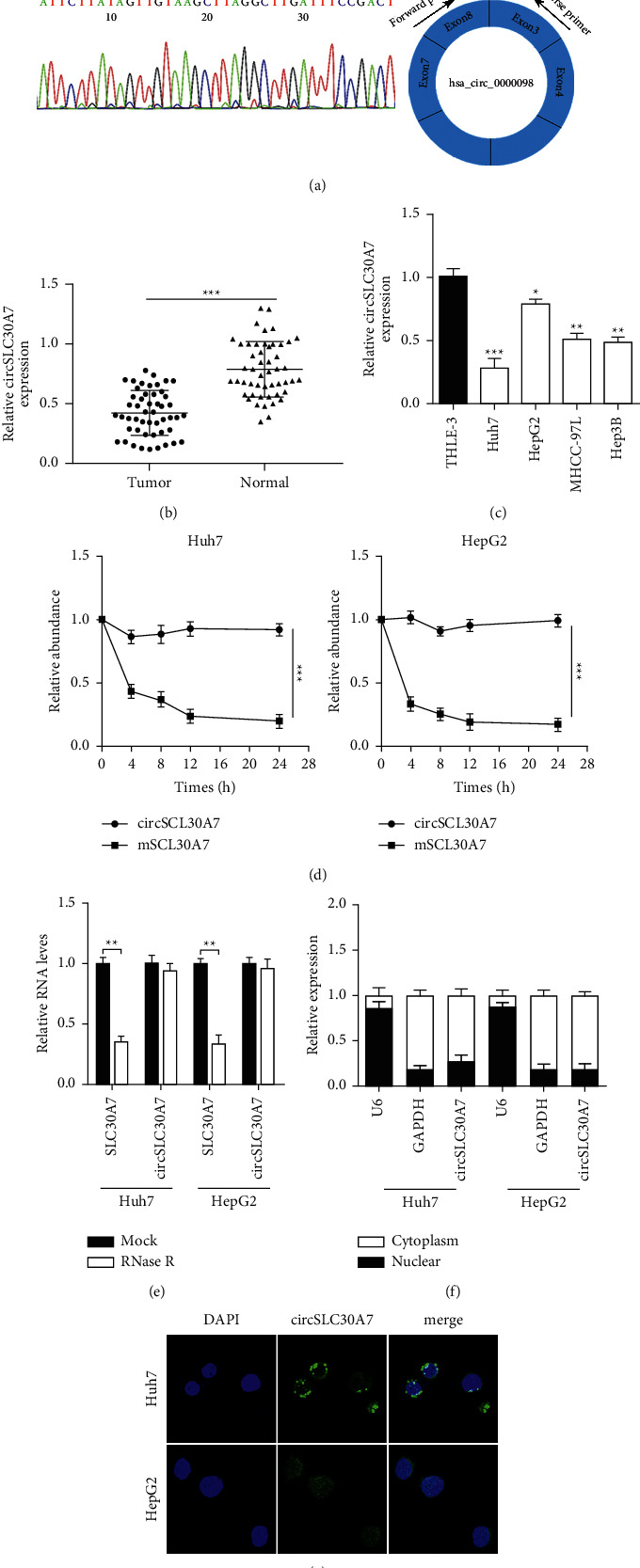
Expression and circRNA characterization of circSLC30A7 in HCC. (a) Sanger sequencing was conducted to confirm head-to-tail splicing. (b) CircSLC30A7 expression in HCC tissues (*n* = 50) and matched normal tissues (*n* = 50) was measured by qRT-PCR. (c) CircSLC30A7 expression in HCC cell lines was detected using qRT-PCR. (d) CircSLC30A7 and SLC30A7 levels were determined after actinomycin D stimulation. (e) CircSLC30A7 and SCL30A7 expression were examined in Huh7 and HepG2 cells exposed to RNase R. (f) CircSLC30A7 was predominantly localized in the cytoplasm by qRT-PCR analysis of cell fractions. (g) CircSLC30A7 was predominantly localized in the cytoplasm by FISH. ^*∗*^*p* < 0.05, ^∗∗^*p* < 0.01, ^∗∗∗^*p* < 0.001.

The analysis of the three GEO databases (GSE78520, GSE94508, and GSE97332) showed that hsa_circRNA_100291 (has_circ_0000098 in circbase) was dow-regulated in HCC (hsa_circRNA_100291 was named as circSLC30A7) (Figure 1(a)). Sanger sequencing was further used for head-to-tail splicing of circSLC30A7. qRT-PCR was used in forcircSL30A7 expression analysis to explore the role of circSLC30A7 in HCC. circSLC30A7 expression was significantly downregulated in 50 pairs of HCC tissues compared to the matched normal samples (Figure 1(b)). circSLC30A7 level in HCC cells was further assessed. circSLC30A7 was substantially decreased in Huh7, HepG2, SMMC-7221, and Bel-7404 than in HL-7702 (Figure 1(c)). Huh7 and HepG2 were selected to examine the stability of circSLC30A7 via RNase R or Actinomycin D stimulation. The Actinomycin D assay showed that circSLC30A7 had a significantly higher half-life than SLC30A7 (Figure 1(d)). RNase R digestion assay indicated that circSLC30A7 was more resistant to RNase R than the linear isoform SLC30A7 (Figure 1(e)). qRT-PCR analysis (Figure 1(f)) and fluorescence in situ hybridization (FISH) (Figure 1(g)) confirmed that circSLC30A7 was located in the cytoplasm. Taken together, these results show that circSLC30A7 is downregulated and stable in HCC.

### 3.2. CircSLC30A7 Inhibits Proliferation and Induces Apoptosis in HCC Cells

**Figure 2:**
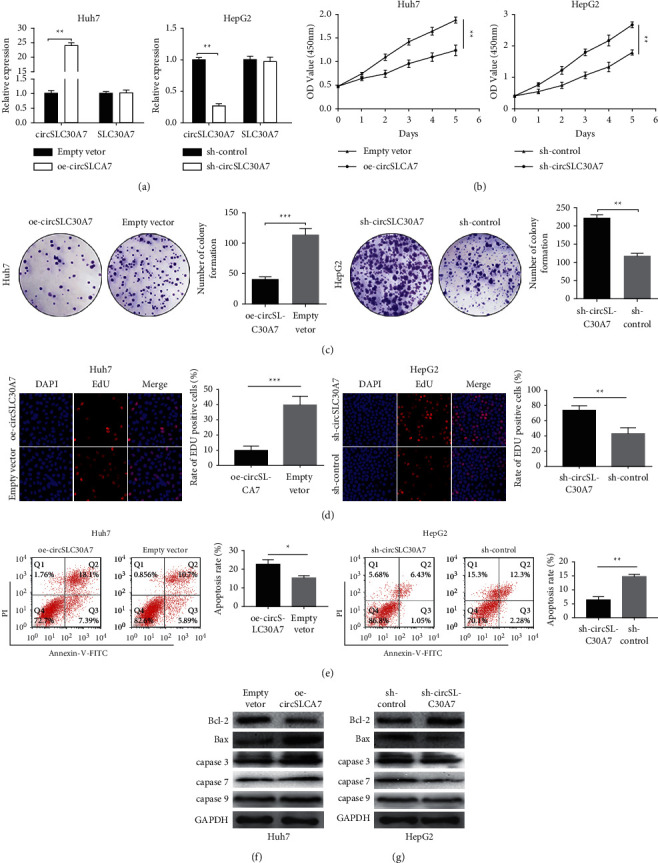
CircSLC30A7 inhibits HCC proliferation *in vitro*. (a) The transfection efficiency of circSLC30A7 was measured using qRT-PCR in Huh7 and HepG2 cells. (b) Cell proliferation ability was assessed by CCK8 assay after transfection *in vitro*. (c) Cell proliferation ability was assessed by colony formation assay after transfection *in vitro*. (d) Cell proliferation ability was assessed by EdU incorporation assay after transfection in vitro. (e) Flow cytometry was used to evaluate the cell apoptosis rate in Huh7 and HepG2 cells. (f and g) The apoptosis-associated protein expression of Bcl-2 and Bax were investigated by western blotting assay in Huh7 cell and HepG2. GAPDH as control. ^*∗*^*p* < 0.05, ^∗∗^*p* < 0.01, ^∗∗∗^*p* < 0.001.

The tumor was more than 5 cm large, and the circSLC30A7 expression was substantially downregulated (Table 1). The circSLC30A7-ov and circSLC30A7-sh were transfected in Huh7 and HepG2, respectively, to investigate the effect of circSLC30A on HCC proliferation using the qRT-PCR assay (Figure 2(a)). CCK8 assay showed that circSLC30A7 overexpression significantly reduced the viability of Huh7 cells while circSLC30A7 downregulation increased the viability of HepG2 (Figure 2(b)). Colony formation assay suggested that circSLC30A7 overexpression significantly decreased the colony numbers of Huh7 cells, while circSLC30A7 knockdown showed increased colony numbers in HepG2 cells (Figure 2(c)). The EdU assays elucidated that the Huh7 cells significantly increased in the circSLC30A7-ov group than the HepG2 cells (Figure 2(d)). Flow cytometry assay was used to evaluate the influence of circSLC30A7 on the apoptosis of HCC cells. The apoptotic rate of HepG2 cells decreased after circSLC30A7-sh transfection while that of Huh7 cells increased (Figure 2(e)). Also, circSLC30A7 overexpression decreased the protein level of Bcl-2 and elevated that of Bax in Huh7 cells (Figure 2(f)), while it showed opposite effects in HepG2 cells (Figure 2(g)). Overall, circSLC30A7 overexpression inhibits proliferation and induces apoptosis in HCC cells.

### 3.3. CircSLC30A7 Directly Targets miR-767-5p

**Figure 3:**
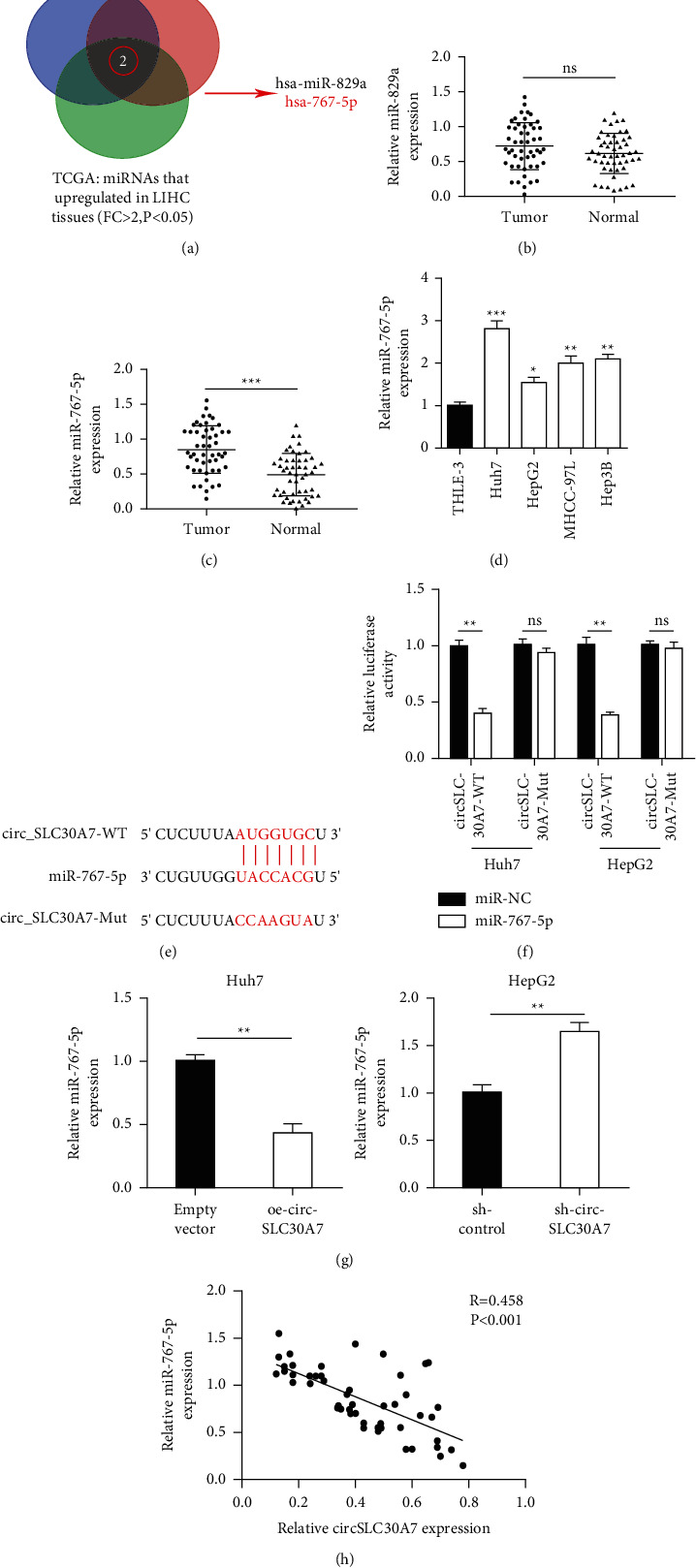
CircSLC30A7 directly targets miR-767-5p. (a) Venn diagram result of three public databases (circBank, circInteractome, TCGA). (b and c) The expression of miR-829a and miR-767-5p was detected in 50 pairs of HCC tissues. (d) The expression miR-767-5p was verified in HCC cell lines by qRT-PCR. (e and f) Luciferase activity was tested Huh7 and HepG2 cells after cotransfection of circSLC30A7-WT or circ-SLC30A7-mut and miR-767-5p-mimic or miR-767-5p-NC. (g) Various transfection of miR-767-5p partially relieved the level of miR-767-5p when altering the expression of circSLC30A7. (h) Spearman's correlation analysis was made to assess the correlation between circSLC30A7 and miR-767-5p. ^*∗*^*p* < 0.05, ^∗∗^*p* < 0.01, ^∗∗∗^*p* < 0.001.

Three public databases (circBank, circInteractome, and TCGA) were used to show that miR-829a and miR-767-5p are the downstream targets of circSLC30A7 (Figure 3(a)). qRT-PCR was then used to confirm the two targets in 50 pairs of HCC tissues. miR-829a expressions were not significantly different in the HCC tissues (Figure 3(b)). However, miR-767-5p expression was substantially upregulated in HCC tissues compared to the normal tissues (Figure 3(c)). Similarly, miR-767-5p expression significantly decreased in Huh7, HepG2, MHCC-97L, and Hep3B compared to THLE-3 cells (Figure 3(d)). CircInteractome (https://circinteractome.nia.nih.gov/) showed that circSLC30A7 and miR-767-5p were related (Figure 3(e)). Therefore, a dual-luciferase reporter experiment was used to assess the relationship. miR-767-5p mimic significantly reduced the luciferase activity of circSLC30A7-WT in Huh7 and HepG2 cells (Figure 3(f)). Besides, ectopic expression of circSLC30A7 regulated miR-767-5p level (Figure 3(g)). Additionally, Pearson's correlation analysis showed that circSLC30A7 expression was negatively correlated with the miR-767-5p level in HCC tissues (Figure 3(h)). Collectively, these results demonstrate that circSLC30A7 directly targets miR-767-5p.

### 3.4. CircSLC30A7 Regulates HCC Proliferation via miR-767-5p Modulation

**Figure 4:**
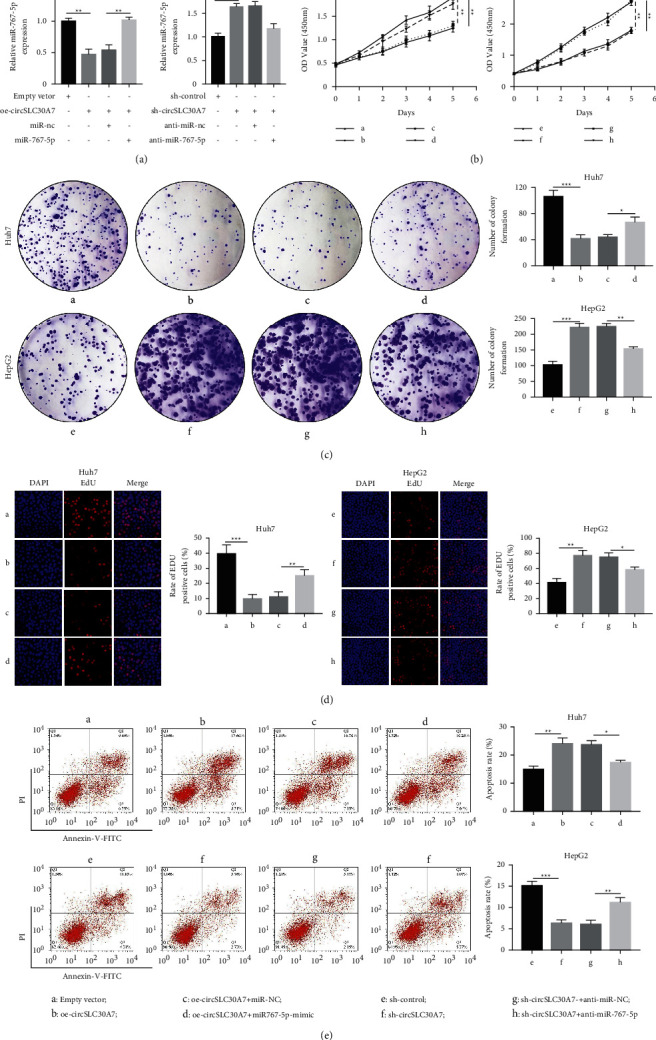
circSLC30A7 inhibits HCC cell proliferation and colony formation and accelerates apoptosis by competitively binding to miR-767-5p. Huh-7 cells were transfected with oe-circSLC30A7 (with empty vector as control) or miR-767-5p mimic + oe-circSLC30A7 (with oe-circSLC30A7 + miR-NC as control). HepG2 cells were transfected with sh-circSLC30A7 (with sh-control as control) or sh-circSLC30A7 + anti-miR-767-5p (with sh-circSLC30A7 +  anti-miR-NC as control). (a) RT-qPCR quantitation of miR-767-5p expression in Huh-7 and HepG2 cells. (b) Proliferation of Huh-7 and HepG2 cells determined by CCK-8 method. (c) Colony formation ability of Huh-7 and HepG2 cells evaluated by colony formation assay. (d) Proliferation of Huh-7 and HepG2 cells determined by EdU incorporation assay. (e) Apoptosis of Huh-7 and HepG2 cells measured by flow cytometry. ^*∗*^*p* < 0.05, ^∗∗^*p* < 0.01, ^∗∗∗^*p* < 0.001. A: empty vector; B: oe-circSLC30A; C: oe-circSLC30A7 + miR--NC; D: oe-circSLC30A7 + miR767-5p-mimic; E: sh-control; F: sh-circSLC30A7; G: sh-circSLC30A7 + anti-miR-NC; H: sh-circSLC30A7 + anti-miR-767-5p.

qRT-PCR analysis showed that miR-767-5p expression substantially increased in HCC cell lines compared with the HL-7702 cell. Therefore, several rescue experiments were conducted by transfecting with Empty vector, oe-circSLC30A7, oe-circSLC30A7 + miR-NC, oe-circSLC30A7 + miR-767-5p-mimic in Huh7 cell and sh-control, sh-circSLC30A7, sh-circSLC30A7 + anti-miR-NC, sh-circSLC30A7 + anti-miR-767-5p in HepG2 cell. Various transfections slightly decreased the miR-767-5p level by altering circSLC30A7 expression (Figure 4(a)). CCK8 assay showed that miR-767-5p mimic partially alleviated the decrease in cell viability induced by oe-circSLC30A7 in Huh7 and HepG2 cells (Figure 4(b)). Colony formation assay and Edu incorporation assay revealed that ectopic expression of circSLC30A7 regulated HCC proliferation by regulating miR-767-5p levels in Huh7 and HepG2 cells (Figures 4(c) and 4(d)). circSLC30A7 overexpression also increased the apoptosis rate of Huh7 cells, which was alleviated by miR-767-5p-mimic. Similarly, circSLC30A7 knockdown reduced the apoptosis rate of HepG2 cells, which was reversed by anti-miR-767-5p (Figure 4(e)). Therefore, circSLC30A7 regulates HCC proliferation via miR-767-5p modulation.

### 3.5. FBXW7 Is the Downstream Target of miR-767-5p

**Figure 5:**
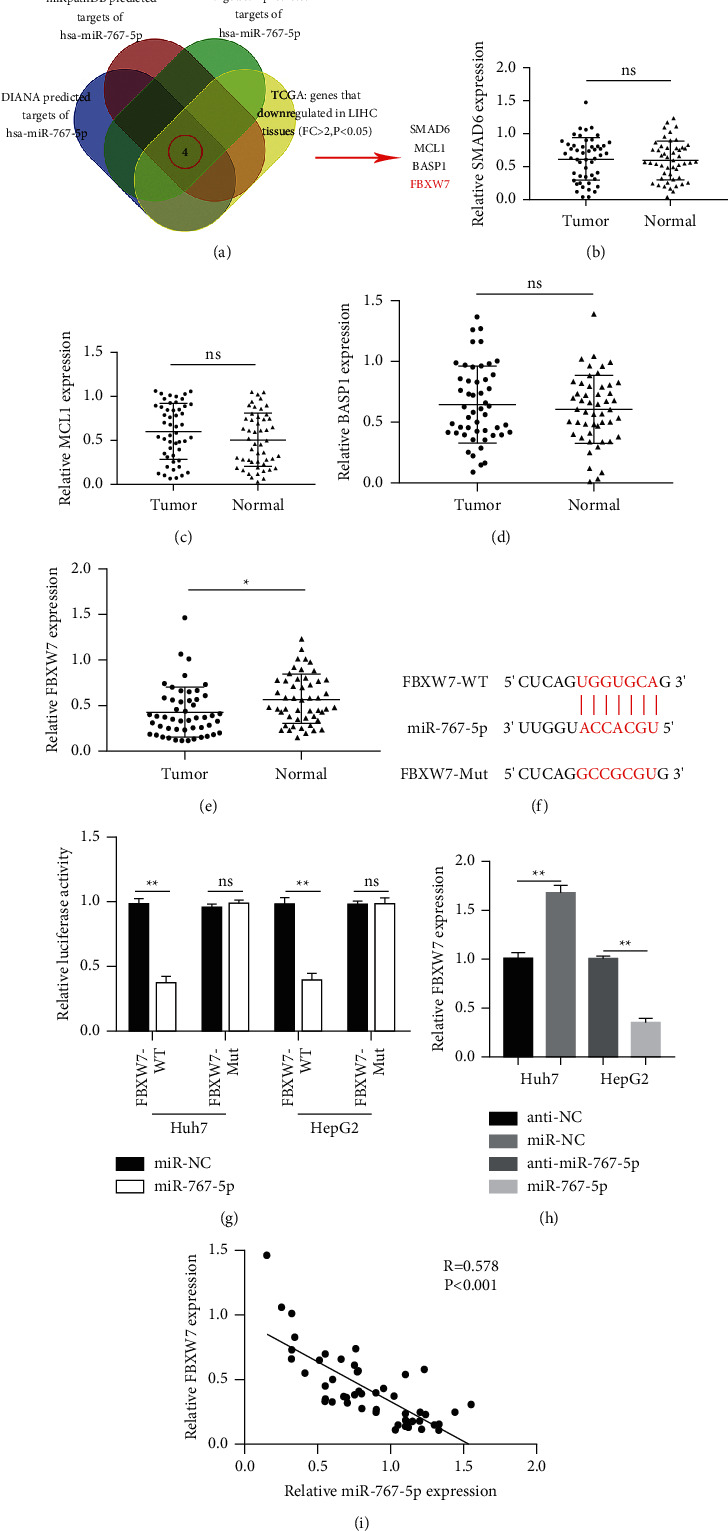
miR-767-5p directly targets FBXW7. (a) Venn diagram result of four online databases (DIANA, miRpathDB, TargetScan, TCGA) showed that there might be four potential target genes of miR-767-5p (SMAD6, MCL1, BASP1, FBXW7). The levels of SMAD6 (b), MCL1 (c), BASP1 (d), and FBXW7 (e) were evaluated in 50 pairs of HCC tissues. (f and g) Luciferase activity was tested Huh7 and HepG2 cells after cotransfection of FBXW7-WT or FBXW-mut and miR-767-5p-mimic or miR-767-5p-NC. (h) The expression of FBXW7 changed with the level of miR-767-5p. (i) Spearman's correlation analysis was used to assess the correlation between miR-767-5p and FBXW7. ^*∗*^*p* < 0.05, ^∗∗^*p* < 0.01.

Venn diagram result of four online databases (DIANA, miRpathDB, TargetScan, TCGA) showed that miR-767-5p has four target genes (SMAD6, MCL1, BASP1, FBXW7) (Figure 5(a)). However, the qRT-PCR analysis indicated that only the FBXW7 level was significantly decreased in HCC tissues compared to the normal samples (Figures 5(b)–5(e)). Moreover, dual-luciferase reported assay indicated that miR-767-5p reduced the luciferase activity of FBXW7-WT in Huh7 and HepG2 cells (Figures 5(f) and 5(g)). FBXW7 expression changed with miR-767-5p levels (Figure 5(h)). Person's correlation analysis demonstrated that miR-767-5p expression was negatively correlated with FBXW7 level in HCC tissues (Figure 5(i)). Overall, these results show that circSLC30A7 modulates FBXW7 through miR-767-5p.

### 3.6. CircSLC30A7 Promotes HCC via NOTCH1 Pathway regulation

**Figure 6:**
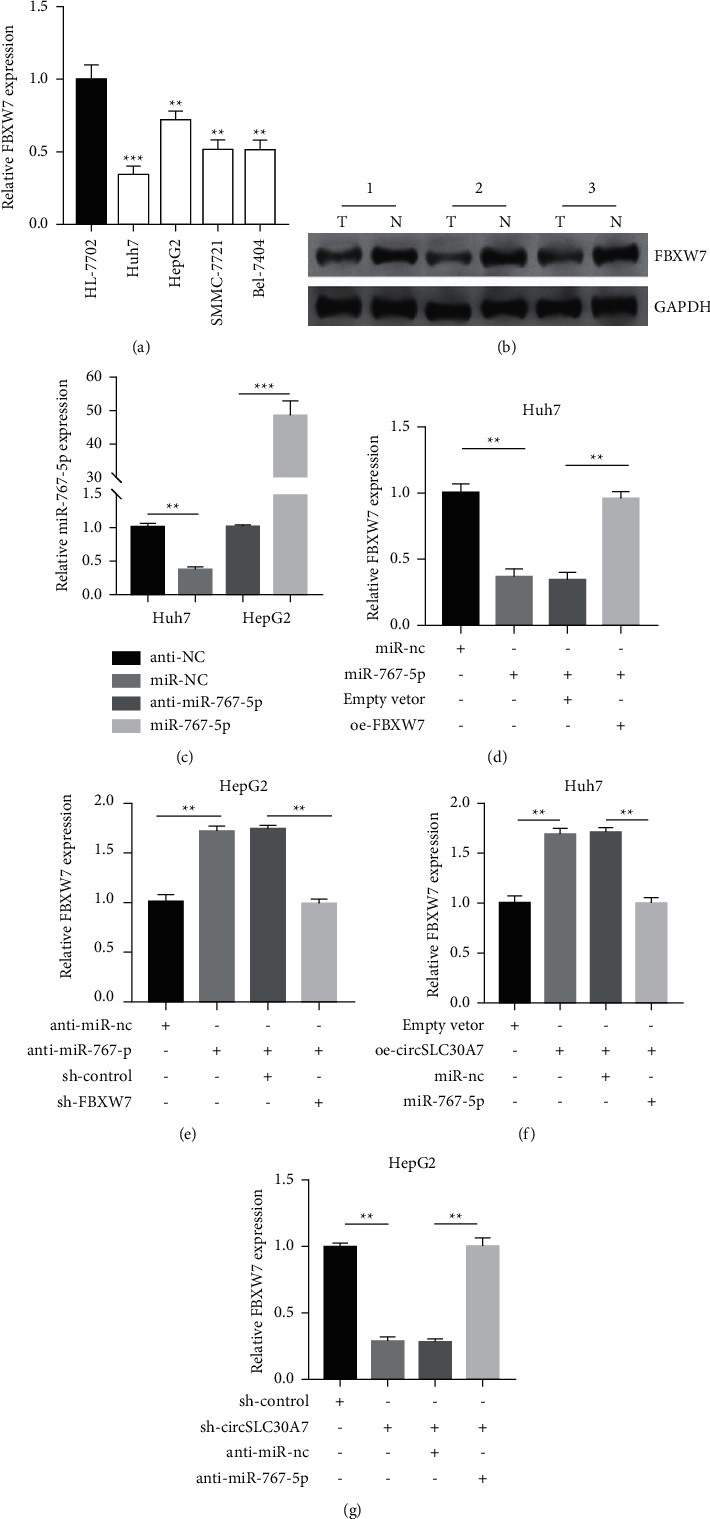
CircSLC30A7 modulates FBXW7 via sponging miR-767-5p. The expression of FBXW7 was measured in HCC cell lines (a) and three pairs of HCC tissues (b). (c) The transfection efficiency of miR-767-5p was measured using qRT-PCR in Huh7 and HepG2 cells. (d–g) The rescue expression experiment was detected after cotransfection in Huh7 and HepG2 cells by utilizing qRT-PCR. ^∗∗^*p* < 0.01, ^∗∗∗^*p* < 0.001.

The qRT-PCR (Figure 6(a)) and western blotting assays showed that FBXW7 levels decreased in HCC cells and three HCC tissues (Figure 6(b)). The transfection efficiency of miR-767-5p in Huh7 and HepG2 cells was then assessed (Figure 6(c)). The transfection of miR-767-5p and FBXW7 partially relieved the decreased FBXW7 expression caused by miR-767-5p overexpression in Huh7 (Figure 6(d)), and HepG2 cells (Figure 6(e)). Besides, circSLC30A7 upregulation increased FBXW7 expression. However, c-transfection of oe-circSLC30A7 and miR-767-5p-mimic reversed the influence in the Huh7 cell (Figure 6(f)). circSLC30A7 silencing repressed FBXW7 expression in HepG2 cell, which was reversed by cotransfection of sh-circSLC30A7 and anti-miR-767-5p (Figure 6(g)).

### 3.7. CircSLC30A7 Promotes HCC via NOTCH1 Pathway regulation

**Figure 7:**
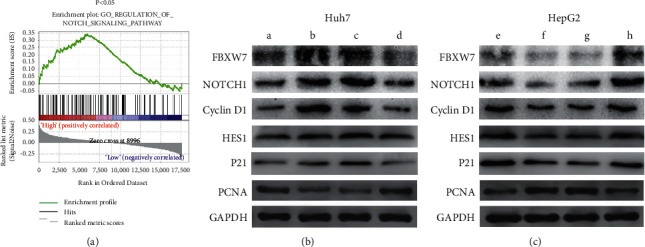
CircSLC30A7 plays role in HCC via regulating the NOTCH1 pathway. (a) Gene Set Enrichment Analysis (GSEA) showed that FBXW7 expression was positively correlated with GO_REGULATION_NOTCH_SIGNALING_PATHWAY. The protein expression levels of FBXW7, NOTCH1, Cyclin D1, and PCNA were detected by western blotting assay in Huh7 cell (b) and HepG2 (c). GAPDH as control. A: empty vector; B: oe-circSLC30A; C: oe-circSLC30A7 + miR--NC; D: oe-circSLC30A7 + miR767-5p-mimic; E: sh-control; F: sh-circSLC30A7; G: sh-circSLC30A7 + anti-miR-NC; H: sh-circSLC30A7 + anti-miR-767-5p.

The Gene Set Enrichment Analysis (GSEA) was used further to determine the underlying mechanism of circSLC30A7 on HCC proliferation. FBXW7 expression was positively correlated with GO_REGULATION_NOTCH_SIGNALING_PATHWAY (Figure 7(a)). Therefore, a western blotting assay was used to assess the protein expression of NOTCH1-associated proteins after cotransfection of circSLC30A7 and miR-767-5p. The circSLC30A7 expression increased the protein level of FBXW7 and decreased that of NOTCH1, Cyclin D1, and PCNA. However, miR-767-5p-mimic alleviated the effect in the Huh7 cell (Figure 7(b)). Similarly, circSLC30A7 silencing reduced the protein level of FBXW7 and amplified that of NOTCH1, Cyclin D1, and PCNA, and the effects were alleviated by anti-miR-767-5p (Figure 7(c)). Therefore, circSLC30A7 inhibits HCC proliferation via the miR-767-5p/FBXW7/NOTCH1 axis.

### 3.8. CircSLC30A7 Inhibits HCC Progression *In Vivo*

**Figure 8:**
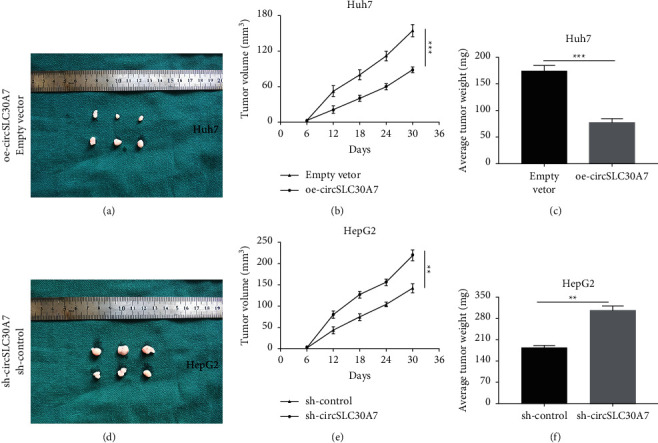
CircSLC30A inhibits HCC cell proliferation *in vivo*. (a–c) Tumor volume and weight were remarkably lower in oe-circSLC30A7 group than those in vector group. (d–f) Tumor volume and weight were evidently elevated in sh-circSLC30A7 group compared with those in sh-control group. ^∗∗^*p* < 0.01, ^∗∗∗^*p* < 0.001.

A xenograft mice model was used to confirm the effect of circSLC30A7 on tumor progression *in vivo*. Tumor volume and weight were substantially lower in the oe-circSLC30A7 group than in the vector group (Figures 8(a)–8(c)). Furthermore, tumor volume and weight were significantly elevated in the sh-circSLC30A7 group compared with the sh-control group (Figure 8(d)–8(f)). Therefore, circSLC30A7 overexpression inhibits HCC progression *in vivo*.

## 4. Discussion

Several studies have reported that circRNAs are essential in the pathogenesis and progression of various cancers. However, fewer studies have investigated the role of circRNAs in HCC. It is necessary to explore the molecular mechanisms of HCC since it is a multistep and multifactorial cancer. Recent reports have shown that several circRNAs are dysregulated in various malignancies, which regulate the initiation and progression of tumors. [16–18] For instance, hsa_circ_0000337, expressed in esophageal squamous cell carcinoma tissues, promotes cell invasion, migration, and proliferation. [19] Circ_0067934 also regulates thyroid carcinoma progression. Therefore, circRNA can be a prognostic biomarker for various malignancies [20].

In this study, circSLC30A7 (circRNA) was used to investigate its role in HCC progression. Herein, circSLC30A7 expression was significantly downregulated in HCC tissues and cells. Low circSLC30A7 expression was associated with the stage and size of the tumor, implying that circSLC30A7 can be a novel biomarker for HCC diagnosis. circSLC30A7 upregulation decreased tumor growth *in vivo*, induced apoptosis, and suppressed colony formation, proliferation, invasion, and migration of HCC cells *in vitro*.

Several studies have also indicated that circRNA containing miRNA binding sites can act as competitive endogenous RNA (ceRNA). Furthermore, some studies have demonstrated that lncRNA and miRNA are associated [21, 22]. Circular RNA AKT3 acts as the sponge of miR-198 and promotes gastric cancer cell proliferation [23]. Circular RNA circTADA2A promotes osteosarcoma cell metastasis and proliferation by sponging miR-203a-3p and modulating CREB3 expression [24]. Similarly, circRNA_100269 is significantly decreased in gastric cancer, inhibiting tumor cell growth [25]. circSLC30A7 can function as a miRNA sponge to decrease HCC malignant phenotypes since it is located in the cytoplasm. In this study, the regulatory mechanism of circSLC30A7 in HCC was assessed. Bioinformatics analysis indicated that CircSLC30A7 was negatively correlated with miR-767-5p in HCC tissues. Luciferase reporter assay indicated that miR-767-5p was a direct target of circSLC30A7 and miR-767-5p knockdown counteracted the tumor-inhibiting activity of circSLC30A7, showing that miR-767-5p is associated with circSLC30A7-mediated cellular senescence in HCC.

Four bioinformatics algorithms were used to predict four potential downstream target genes of the circSLC30A7/miR-767-5p axis in HCC. Further experiments revealed that FBXW7 was the downstream effector of the circSLC30A7/miR-767-5p axis and mediated cellular senescence in HCC. FBXW7 is a tumor suppressor [26]. However, FBXW7 and miR-767-5p were negatively correlated in HCC tissues. The protein level of FBXW7 mRNA decreased in HCC tissues. qRT-PCR and western blot showed that circSLC30A7 negatively regulated FBXW7 via the miR-767-5p. Besides, the GSEA assay and western blotting assay indicated that the NOTCH1 signaling pathway was associated with circSLC30A7.

In conclusion, circSLC30A7 (ceRNA) induces FBXW7 via miR-767-5p. Importantly, circSLC30A7 upregulation inhibited HCC proliferation via miR-767-5p/FBXW7/NOTCH1 axis *in vitro* and *in vivo*. Therefore, this study provides promising strategies for HCC therapy.

## Figures and Tables

**Table 1:** The relationship between the expression of circSLC30A7 and the clinical characteristics of HCC patients.

Characteristics	Number	circSLC30A7 expression		*P* value
		High group	Low group	
*Age (years)*				
<60	14	5	9	0.207
≥60	36	20	16	

*Gender*				
Male	27	13	14	0.776
Female	23	12	11	

*Size (cm)*				
<5	23	15	8	0.047^*∗*^
≥5	27	10	17	

*Stage*				
I/II	21	14	7	0.044^*∗*^
III/IV	29	11	18	

*Differentiation grade*				
Well + moderate	26	13	13	0.999
Poor	24	12	12	

*Metastasis*				
Absent	28	15	13	0.568
Present	22	10	12	

^*∗*^ *p* < 0.05, statistically significant difference.

## Data Availability

The data used to support the findings of this study are available from the corresponding author upon request.
